# Effects of deliberate practice at different training frequencies on long-term retention of high-quality chest compression proficiency among novice nurses: a quasi-experimental study

**DOI:** 10.3389/fmed.2026.1751992

**Published:** 2026-04-10

**Authors:** Liqun Huang, Chuanren Ling, Weisheng Chen, Rongman Li, Wei Zhang, Ping Cao, Xiaoyu Liao, Xiaoyan Lin, Banghan Ding, Qiuying Deng

**Affiliations:** 1 The Second Affiliated Hospital of Guangzhou University of Chinese Medicine, Guangzhou, Guangdong, China; 2 The Second Affiliated Hospital of Guangzhou University of Chinese Medicine, Guangzhou, China

**Keywords:** basic life support, cardiopulmonary resuscitation, chest compression, deliberate practice, high-quality CPR, novice nurses

## Abstract

**Background:**

Nurses are pivotal first responders in in-hospital cardiac arrest (IHCA), and timely, guideline-concordant BLS substantially improves survival. While deliberate practice (DP) enhances skill acquisition, the optimal retraining interval for maintaining long-term proficiency among novice nurses remains unclear. This study aimed to compare the effects of single versus repeated DP sessions on the 6-month retention of Cardiopulmonary Resuscitation (CPR) quality and knowledge.

**Methods:**

A quasi-experimental study enrolled 148 novice nurses allocated to Single-DP group (baseline training only) a Repeated-DP group (baseline plus a 3-month booster session). CPR quality (compression depth, rate, chest recoil, compression fraction), theoretical knowledge, and overall skill scores were assessed at baseline, immediately after training, and 6-month follow-up. Generalized estimating equations (GEE) assessed group, time, and their interaction.

**Results:**

A total of 148 novice nurses completed the study, with no significant differences in baseline characteristics. Immediately after training, the Single-DP group achieved higher scores in theoretical knowledge (*P* = 0.001) and overall skill (*P* = 0.011). At the 6-month follow-up, the Repeated-DP group demonstrated superior retention in raw performance metrics, maintaining significantly greater median compression depth (52.00 vs. 47.50 mm; *P* = 0.014) and higher overall skill scores (57.72 vs. 54.55; *P* < 0.001). However, GEE analysis revealed no significant group-by-time interaction at 6 months for knowledge (*P* = 0.344) or overall skill (*P* = 0.400), suggesting that the repeated intervention did not yield a statistically significant benefit in adjusted retention rates. Notably, prior real-world CPR experience was identified as a significant predictor of skill retention (*P* = 0.004).

**Conclusion:**

DP significantly improves CPR skill proficiency in novice nurses. However, a 3-month refresher did not enhance 6-month retention compared to single training. The finding that prior real-world experience significantly predicts retention underscores the gap between simulation and clinical practice. Therefore, to ensure high-quality patient care, CPR training programs should replace single-session instruction with high-frequency, high-fidelity simulation-based education for novice nurses.

## 1 Introduction

Cardiac arrest remains a significant global health burden, with hundreds of thousands of individuals experiencing cardiac arrest annually (1, 2). In-hospital cardiac arrest (IHCA) is particularly prevalent and associated with low survival-to-discharge rates. In the United States alone, an estimated 290,000 IHCAs occur each year, with an incidence ranging from 1.2 to 9–10 cases per 1,000 hospital admissions (3). Timely and high-quality cardiopulmonary resuscitation (CPR) is a critical determinant of survival and favorable neurological outcomes for patients experiencing IHCA (4–7).

Nurses play a pivotal role in the IHCA response chain, serving as first responders at the bedside, key members of resuscitation teams responsible for identifying and managing early warning signs, and often as clinical or administrative leaders during emergencies. As such, patient prognosis is heavily dependent on the quality of basic life support (BLS) delivered by nurses (8, 9). Therefore, proficiency in effective BLS is an essential competency for all clinical nurses. Despite mandatory CPR training, healthcare professionals—including nurses—frequently fail to deliver CPR that meets performance standards set by major guidelines such as those of the American Heart Association (AHA) and the European Resuscitation Council (ERC) (10, 11). This challenge is especially pronounced among novice nurses, who lack real-world emergency experience. Although nursing curricula include BLS instruction, many graduates have not fully mastered the necessary knowledge and psychomotor skills and receive insufficient ongoing training to maintain competence over time (12). Consequently, novice nurses often enter clinical practice inadequately prepared to perform high-quality CPR.

Conventional BLS courses have long served as the standard training model but are increasingly recognized as suboptimal due to poor long-term skill retention and high resource demands (13). Evidence indicates that these courses do not reliably ensure skill acquisition, often lack simulation-based practice, real-time feedback mechanisms, and objective performance assessment tools, and lack evidence-based recommendations for retraining frequency (14, 15). Without repeated, deliberate practice, CPR skills begin to deteriorate within weeks of initial training (16). To address these limitations, the American Heart Association’s Resuscitation Quality Improvement (RQI) program recommends frequent interval training—short, structured CPR practice sessions with skills assessment at least every 3 months—for healthcare providers. Prior studies support this approach, demonstrating that more frequent BLS training intervals enhance both skill development and retention across diverse healthcare populations (17–20). These findings suggest a positive association between training frequency and skill maintenance. However, existing research has notable limitations: some studies suffer from small sample sizes, while others focus on experienced nurses with prior BLS/Advanced Cardiac Life Support (ACLS) certification or on student populations such as nursing students (21, 22). As a result, their findings may not be generalizable to novice nurses entering clinical practice.

Moreover, CPR training is physically demanding, and excessive repetition without individualization may lead to fatigue, reduced motivation, and diminished training effectiveness. Emerging evidence suggests that personalized training schedules could improve performance consistency while reducing overall training time and burden (23). Despite this, the training needs of novice nurses remain underexplored, and there is a paucity of data on optimal interval training strategies specifically tailored to this population. This study is grounded in Ericsson’s theory of deliberate practice, which posits that expert-level performance is developed through structured, repetitive engagement in targeted activities accompanied by immediate and informative feedback (24). Unlike simple repetition, deliberate practice (DP) involves focused efforts to refine specific components of performance, typically achieved by decomposing complex skills into manageable elements. In the context of CPR training, this entails deliberate rehearsal of critical technical aspects—such as chest compression depth and rate—guided by real-time performance feedback. Skill decay remains a well-documented challenge in CPR training.

We hypothesized that more frequent intervals of DP (every 3 months) are better aligned with the principles of sustained skill acquisition and retention. By delivering reinforcement prior to substantial skill degradation, these shorter intervals should facilitate stronger schema consolidation and slower deterioration compared to a single training session. Specifically, repeated practice at 3-month intervals may promote greater automation of procedural memory and support durable learning through cumulative encoding. So we conducted a quasi-experimental trial comparing overall CPR skill quality between novice nurses receiving repeated DP training at 3-month intervals and those receiving a single session of deliberate practice. The primary objective was to determine the optimal training frequency for maximizing CPR skill retention among novice nurses.

## 2 Materials and methods

### 2.1 Study design

A quasi-experimental study design was utilized to achieve the aim of this study. This study employed a prospective two-cluster randomized allocation design. Due to practical and operational constraints inherent in randomizing nurses at the individual level within real-world clinical environments, eligible nurses from the Guangdong Provincial Hospital of Chinese Medicine (Main Hospital and University City Branch) were aggregated into one cluster, and those from the Ersha Island Branch, Fangcun Branch into the other. Cluster assignment was conducted via computer-generated randomization, allocating one cluster to the Repeated-Deliberate Practice group and the other to the Single-Deliberate Practice group. Given the limited number of clusters, the design carried an inherent risk of selection bias. To mitigate potential confounding, we systematically collected and compared baseline characteristics—including hospital size, departmental structure, nurse demographics (age, gender, years of service), and prior frequency of emergency training—to rigorously assess intergroup comparability and strengthen the validity of the observed outcomes.

### 2.2 Study population and sample size

Participants were registered nurses within 2 years of initiating clinical practice following graduation, and were not required to hold current certification in BLS or ACLS. Individuals unable to perform chest compressions for medical or physical reasons were excluded. All participants received comprehensive written and verbal information about the study’s objectives, procedures, and potential risks, and provided written informed consent prior to enrollment.

The sample size was estimated based on the primary outcome measure. A previous study showed that the proportion of “excellent CPR” in the 6-month group was less than 21%. We expected to detect a 25% difference in the proportion of excellent CPR between groups, with a significance level of 0.05 and a power of 0.8. The sample size ratio of the two groups was 1:1 and was calculated using the following formula:


N=

$$\frac{\begin{array}{c} {}[Z_{a/2}\sqrt{2\,\bar{p}\,\left(1-\bar{p}\right)\,\left(Q_{1}^{-1}+Q_{2}^{-1}\right)}\\ +Z_{\beta }\sqrt{p_{1}\,\left(1-p_{1}\right)/Q_{1}+p_{2}\,\left(1-p_{2}\right)/Q_{2}}]{}^{2} \end{array}}{{\left(p_{1}-p_{2}\right)}^{2}}$$

Finally, to accommodate an anticipated 10% attrition rate, the target sample size was adjusted to 77 participants per group.

### 2.3 Intervention

The study was conducted in a dedicated clinical simulation laboratory to ensure environmental consistency. To minimize instructional variability, the intervention followed a rigorous, standardized protocol aligned with the 2020 AHA Guidelines for CPR and Emergency Cardiovascular Care (ECC).

#### 2.3.1 Instructor standardization

To ensure implementation fidelity and rigorous quality assurance, a Core Quality Oversight Committee was established. This committee consisted of one head nurse and three supervising nurses, two of whom held active AHA certified BLS/ACLS instructor credentials. All training instructors met prespecified eligibility criteria: (1) ≥10 years of clinical nursing experience; (2) successful completion of an AHA-endorsed instructor training curriculum; and (3) active certification as BLS/ACLS instructors. Prior to study initiation, all instructors attended a standardization workshop designed to align instructional delivery, skill assessment protocols, and real-time performance feedback practices to minimize instructor variability.

#### 2.3.2 Training procedure

Participants in the intervention group underwent a structured training program consisting of:

Baseline assessment: Before training, novice nurses completed a demographic questionnaire (covering physical characteristics, clinical experience, and prior CPR training) and performed a 2-min CPR skills test without real-time feedback to establish baseline proficiency.

Theoretical instruction: A 45-min didactic session covering the chain of survival, high-quality CPR metrics, and AED usage.

Expert demonstration: A certified instructor performed a 2-min high-quality CPR demonstration to model the correct technique.

DP with real-time feedback: Hands-on training followed the DP model with a 1:6 instructor-to-student ratio. Participants practiced on Resusci Anne QCPR manikins (Laerdal Medical, Stavanger, Norway) connected to feedback devices. This setup provided immediate visual and auditory feedback on compression depth, rate, and recoil, allowing for instant self-correction. Instructors monitored performance via data from these devices and provided immediate, individualized guidance.

Feedback loop: After each cycle, instructors provided direct feedback—positive reinforcement for correct actions and corrective guidance for deviations. Trainees repeated the practice until they achieved mastery of the entire CPR process or the session time expired (approximately 2 h).

**FIGURE 1:**
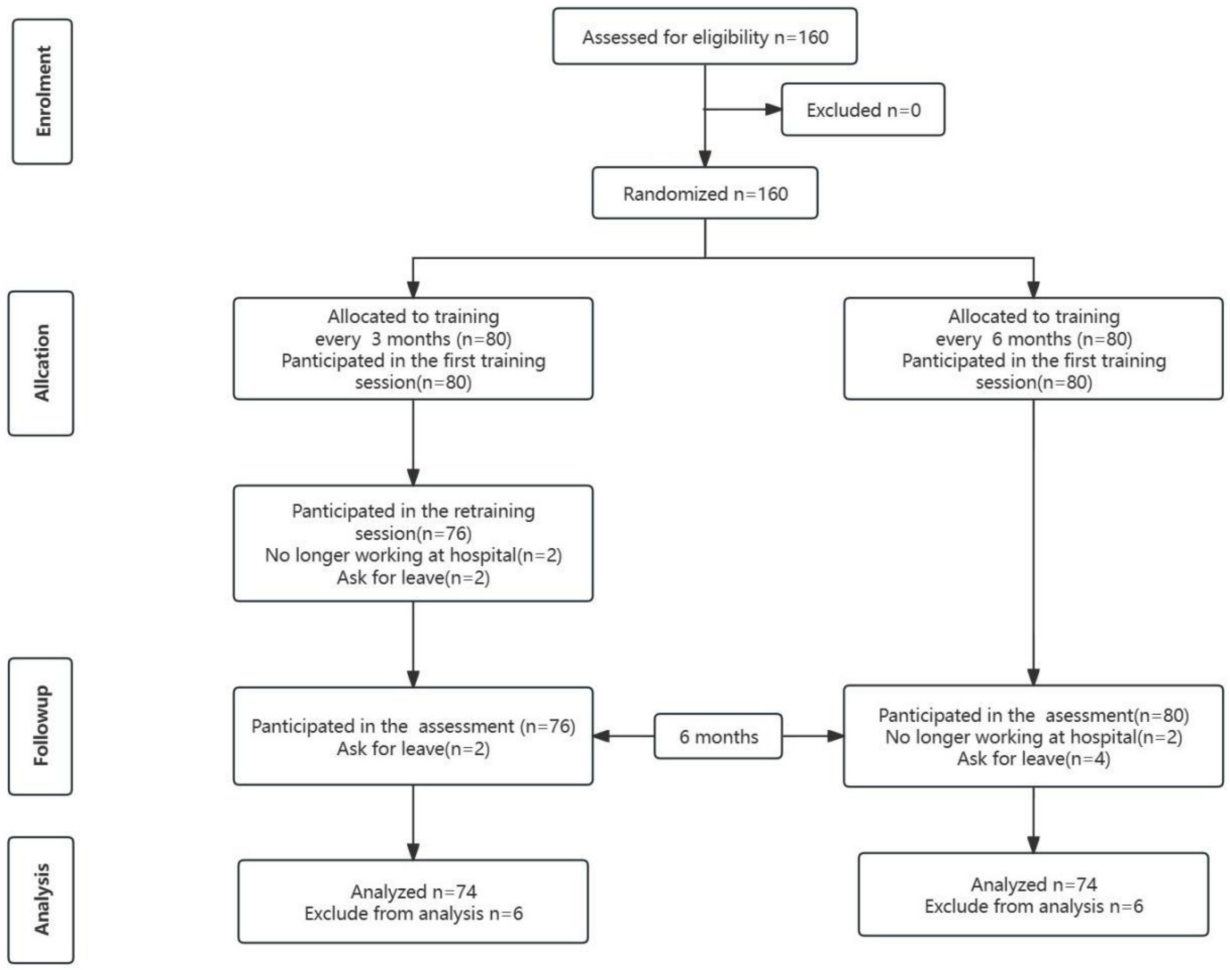
Participant flow chart. Consort flow-chart for comparing Single-DP training with Repeated-DP training for high-quality cardiopulmonary resuscitation skills in a quasi-experimental study.

Group design: Both groups received identical initial training. The Repeated-DP group received an additional booster training session 3 months after the initial training, the Single-DP group only received one training session (as detailed in Figure 1). Outcomes were assessed at baseline, immediately post-training, and 6-month follow-up.

### 2.4 Data collection

The data collection instruments comprised four complementary components: (1) a sociodemographic questionnaire; (2) a theoretical knowledge assessment; (3) a structured observational skills checklist administered during manikin-based simulation; and (4) sensor-based CPR overall skills metrics.

The theoretical knowledge (Supplementary Material 1) was assessed using 20 closed-ended multiple-choice questions (MCQs) aligned with the 2020 AHA Guidelines for Cardiopulmonary Resuscitation and Emergency Cardiovascular Care (25, 26). Optimal score was 100 (five points per correct answer); higher scores reflected greater theoretical knowledge. Consistent with the AHA established passing standard for BLS certification—which requires a minimum performance accuracy of 80%—participants scoring ≥80 were classified as having achieved competency, whereas those scoring <80 were deemed not yet competent in BLS skills. The Cronbach’s α was 0.702, indicating acceptable internal consistency.

The Structured Skill Checklist was prepared following an extensive review of the available literature such as Berden et al. (27), Graham (28), while also referring to the Directly Observed Procedural Skills assessment (DOPS) scoring system (29). It was designed and administered to assess participants’ BLS performance (Supplementary Material 2). The checklist comprises eight critical BLS steps: scene safety; responsiveness assessment; emergency response activation; correct hand placement and body positioning for compressions; compression rate (100–120/min); full chest recoil between compressions; appropriate airway opening; and two effective ventilations with visible chest rise. The maximum possible score was 74, with higher scores indicating better BLS skills. A cut-off of 80% (score ≥ 60) was established to define competency, aligning with the mastery learning standard recommended by AHA for Basic Life Support training. Internal consistency was confirmed by a Cronbach’s α coefficient of 0.929.

For objective metric collection: Participants performed a 2-min adult CPR sequence on a high-fidelity manikin (Resusci Anne SkillGuide, Laerdal Medical) with Palm CPR feedback sensors (Link CPR, SunLife Science). The system automatically recorded the percentage of compressions meeting guideline targets: depth (50–60 mm), rate (100–120/min), and full chest recoil. Secondary outcomes were mean depth (mm), rate (min^−1^), and chest compression fraction (CCF).

### 2.5 Statistical analysis

Descriptive statistics are presented as frequencies and percentages for categorical variables, and as means ± standard deviations (SD) for continuous variables with normal distributions. Non-normally distributed continuous variables are reported as medians and interquartile ranges (IQR). Baseline between-group comparisons of categorical variables were performed using the chi-square test or Fisher’s exact test, as appropriate. Between-group differences at follow-up were analyzed using the Mann–Whitney U test. Generalized estimating equations (GEE) were applied to evaluate the longitudinal effects of group and time on skill retention. All analyses were performed using IBM SPSS Statistics 22.0 (Armonk, NY: IBM Corp., 2013). A two-sided *p*-value < 0.05 was considered statistically significant. Figures were created using GraphPad Prism 8.1.1 (GraphPad Software, La Jolla, CA).

## 3 Results

### 3.1 Socio-demographic characteristics

A total of 160 participants were enrolled; 12 were excluded due to loss to follow-up (four no longer employed at the hospital, eight withdrawn for administrative reasons), yielding a final analytic sample of 148 participants who completed the training protocol. The sample consisted of 23 males (15.5%) and 125 females (84.5%), with a mean age of 23.0 years (SD = 1.4). The majority were employed in general medical wards (62, 41.9%). During training, 39.2% of participants used CPR feedback devices; 67.6% had received CPR training within the past year; and only 7.4% had previously performed chest compressions during actual cardiac arrest events. No significant baseline differences were found between groups in demographics or professional characteristics (Table 1).

**TABLE 1:** Baseline comparison of sociodemographic and work-related factors between the Repeated-DP and the Single-DP group (*N* = 148).

Variables	Total	Single-DP group (*n* = 74)	Repeated-DP group (*n* = 74)	χ^2^/t	*P*-value
Gender, *n* (%)					
Female	23 (15.54)	12 (52.17)	11 (47.83)	0.051^b^	0.821
Male	125 (84.46)	62 (49.60)	63 (50.40)	–	–
Age, years, mean (SD)	23.40 (1.36)	23.22 (1.04)	23.58 (1.61)	−1.643^a^	0.103
BMI, *n* (%)					
Underweight	36 (24.66)	15 (20.55)	21 (28.77)	1.586^b^	0.453
Normal weight	99 (66.81)	53 (72.60)	46 (63.01)	–	–
Overweight or obesity	11 (7.53)	5 (6.85)	6 (8.22)	–	–
Educational qualifications, *n* (%)					
Junior college	74 (50.00)	37 (50.00)	37 (50.00)	0.000^b^	1.000
Udergraduate	74 (50.00)	37 (50.00)	37 (50.00)	–	–
Location of work, *n* (%)					
Emergency room	26 (17.57)	17 (22.97)	9 (12.16)	3.899^b^	0.564
Intensive care unit	14 (9.46)	7 (9.46)	7 (9.46)	–	–
Cardiovascular ward	17 (11.49)	7 (9.46)	10 (13.51)	–	–
Surgical ward	16 (10.81)	9 (12.16)	7 (9.46)	–	–
Orthopedic ward	13 (8.78)	6 (8.11)	7 (9.46)	–	–
General medical ward	62 (41.89)	28 (37.84)	34 (45.95)	–	–
CPR feedback device used on simulated patient, *n* (%)					
Yes	58 (39.19)	27 (36.49)	31 (41.89)	0.454^b^	0.501
No	90 (60.81)	47 (63.51)	43 (58.11)	–	–
CPR training in the past year, *n* (%)					
Yes	100 (67.57)	45 (60.81)	55 (74.32)	3.083^b^	0.079
No	48 (32.43)	29 (39.19)	19 (25.68)	–	–
Prior experience with chest compression on real patient, *n* (%)					
Yes	11 (7.43)	5 (6.76)	6 (8.11)	0.098^b^	0.754
No	137 (92.57)	69 (93.24)	68 (91.89)	–	–

SD, standard deviation.

*^a^*Independent-samples *t*-test.

*^b^*Chi-square test. *Variable with *p* < 0.05.

### 3.2 Comparison of chest compression quality between the two groups across time points

Table 2 presents CPR quality metrics and competency scores across three time points: baseline (pre-training), immediately after training (T1), and 6-month follow-up (T2). At baseline, the Single-DP and Repeated-DP groups had comparable compression depth, rate, recoil, CCF, knowledge, and skill scores (all *p* > 0.05).

**TABLE 2:** Comparison of chest compression quality between the two groups across time points (pre-training, immediately after training, 6-month follow-up).

Variable	Group	Pre-training	immediately after training	6-month follow-up	Difference
Percentage of correct compression depth (%)	Single-DP	11.49 (1.44, 34.70)	37.81 (18.37, 47.94)	34.22 (16.08, 50.23)	−4.72 (−19.10, 14.59)
	Repeated-DP	18.60 (1.30, 43.66)	43.53 (17.08, 65.77)	39.14 (25.45, 48.94)	−4.45 (−29.50, 22.10)
	*Z*	−0.730	−1.355	−1.651	−0.067
	*P*-value	0.465	0.175	0.098	0.946
Percentage of correct compression depth rate (%)	Single-DP	43.97 (3.99, 81.71)	67.41 (52.50, 90.01)	81.03 (0.2848, 0.9459)	−1.01 (−25.70, 25.46)
	Repeated-DP	62.95 (14.81, 89.97)	64.15 (60.45, 91.51)	76.89 (0.3518, 0.9517)	1.64 (−28.62, 27.01)
	*Z*	−1.703	−0.476	−0.027	−0.470
	*P*-value	0.088	0.634	0.980	0.639
Percentage of complete chest recoil (%)	Single-DP	100.00 (98.61, 100.00)	100.00 (100.00, 100.00)	100.00 (100.00, 100.00)	0.00 (0.00, 0.00)
	Repeated-DP	100.00 (99.45, 100.00)	100.00 (86.73, 100.00)	100.00 (100.00, 100.00)	0.00 (0.00, 0.13)
	*Z*	−0.634	−3.624	−0.025	−2.679
	*P*-value	0.526	<0.001*	0.980	0.007*
Compression depth (mm)	Single-DP	43.00 (39.00, 48.75)	47.00 (43.00, 49.00)	47.50 (43.00, 53.00)	2.50 (−3.00, 6.00)
	Repeated-DP	45.00 (39.00, 50.00)	46.00 (40.25, 51.00)	52.00 (46.00, 57.75)	5.00 (−3.00, 12.00)
	*Z*	−0.823	−0.326	−2.458	−2.010
	*P*-value	0.410	0.744	0.014*	0.044*
Compression rate (per minute)	Single-DP	110.50 (98.50, 119.75)	116.00 (110.25, 121.75)	111.50 (106.00, 119.50)	−5.00 (−10.00, 6.00)
	Repeated-DP	109.00 (102.00, 117.75)	118.00 (111.00, 123.00)	111.00 (106.00, 118.75)	−5.00 (−12.00, −0.25)
	*Z*	−0.140	−1.056	−0.012	−0.885
	*P*-value	0.889	0.291	0.991	0.376
CCF	Single-DP	48.00 (41.25, 53.00)	51.00 (47.00, 55.00)	50.50 (44.00, 56.00)	0.00 (−6.00, 5.25)
	Repeated-DP	44.00 (39.00, 50.75)	50.00 (47.00, 54.00)	51.50 (48.00, 57.00)	2.00 (−4.00, 7.00)
	*Z*	−1.722	0.402	−1.384	−1.519
	*P*-value	0.085	0.688	0.166	0.128
Knowledge score, score	Single-DP	75.00 (65.00, 85.00)	90.00 (80.00, 100.00)	69.26 (60.000, 75.000)	−20.00 (−35.00, −5.00)
	Repeated-DP	70.00 (60.00, 80.00)	81.31 (75.00, 90.00)	70.00 (60.00, 75.00)	−15.00 (−25.00, 0.00)
	*Z*	−1.791	−3.388	−0.334	−2.213
	*P*-value	0.073	0.001*	0.738	0.027*
Overall skills, score	Single-DP	48.40 (42.50, 54.18)	62.55 (58.95, 66.40)	54.55 (48.35, 58.18)	−7.75 (−12.43, −3.60)
	Repeated-DP	45.90 (36.83, 53.88)	58.50 (53.18, 64.38)	57.72 (56.65, 61.88)	−1.18 (−6.78, 4.45)
	*Z*	−1.528	−2.556	−3.576	−4.729
	*P*-value	0.126	0.011*	<0.001*	<0.001*

*Variable with *p* < 0.05.

Immediately following training, both groups demonstrated improvements. The Single-DP group scored higher in knowledge (90.00 vs. 81.31; *p* = 0.001) and overall skill (62.55 vs. 58.50; *p* = 0.011). Recoil was higher in the Single-DP group (*p* < 0.001), though both achieved 100% median recoil. No differences emerged for compression depth, rate, or CCF.

**FIGURE 2:**
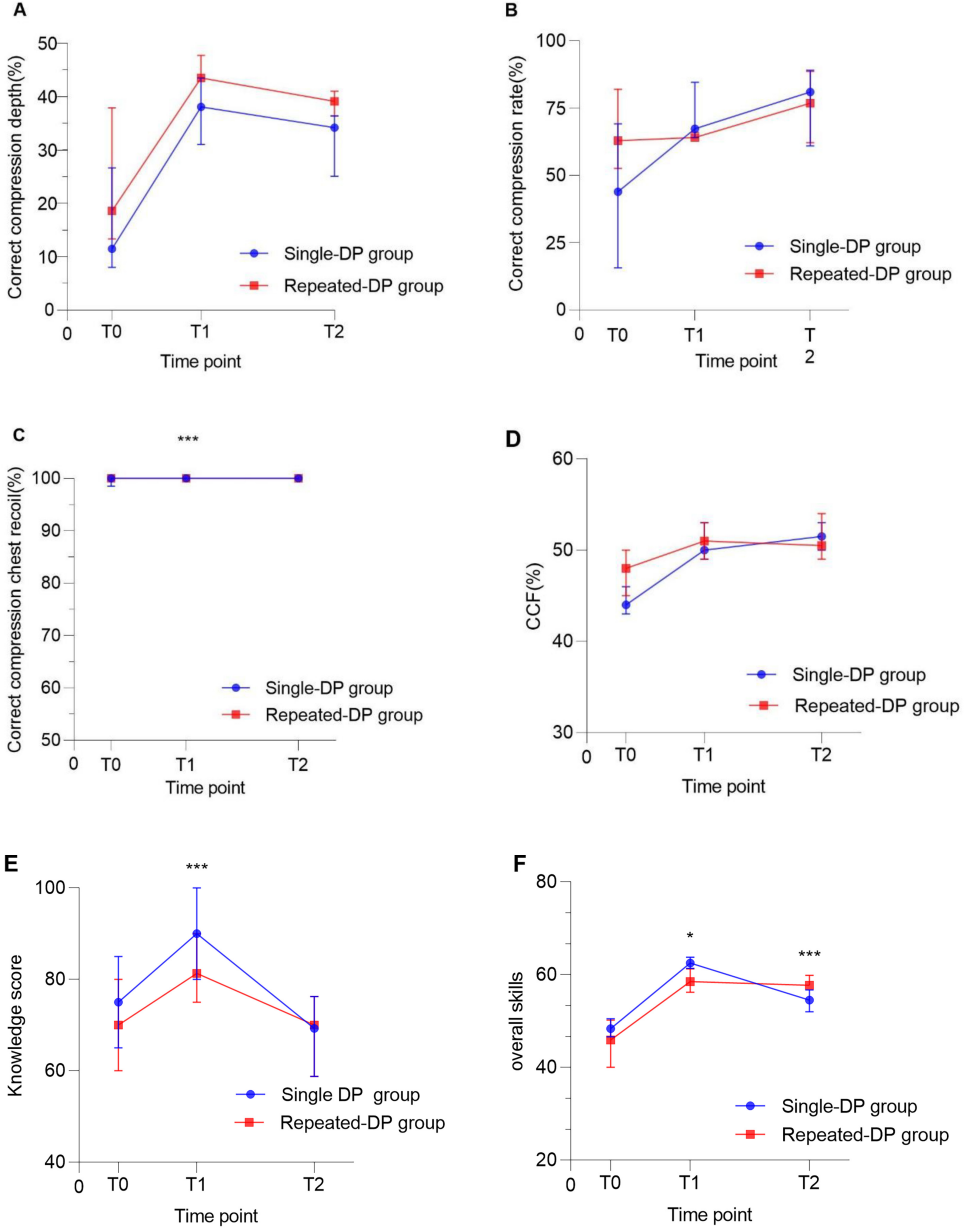
Trends in guideline-concordant chest compression (depth, rate, recoil, chest compression fraction), theoretical knowledge, and overall skill scores across two groups at three time points: pre-training, immediately post-training, and 6 months post-training. **(A)** Trends in percentage of correct compression depth. **(B)** Trends in percentage of correct compression rate. **(C)** Trends in percentage of correct compression chest recoil. **(D)** Trends in CCF. **(E)** Trends in knowledge scores. **(F)** Trends in overall skills scores. T0, pre-training; T1, immediately after training; T2, 6-month follow-up. *Variable with *p* < 0.05; ***Variable with *p* < 0.001.

At the 6-month follow-up (T2), the Repeated-DP group maintained significantly greater compression depth (52.00 vs. 47.50 mm; *p* = 0.014) and overall skill scores (57.72 vs. 54.55; *p* < 0.001) relative to the Single-DP group. Analysis of the “Difference” (T2 minus T1) showed the Repeated-DP group had less overall skill decay (−1.18 vs. −7.75; *p* < 0.001), less knowledge decay (−15.00 vs. −20.00, *p* = 0.027), and greater compression depth gain (+5.00 vs. +2.50 mm, *p* = 0.044). Compression quality declined over time in both groups (Figure 2).

### 3.3 Effectiveness of the Repeated-Deliberate Practice group versus the Single-DP group in sustaining CPR knowledge retention across assessment time points (pre-training, immediately after training, and 6-month follow-up) adjusted with covariates

Table 3 presents the adjusted comparative effectiveness of the Repeated-Deliberate Practice group and the Single-DP group on CPR knowledge retention across three standardized assessment time points—pre-training (T0), immediate post-training (T1), and 6-month follow-up (T2)—after controlling for covariates (age, gender, and BMI). A significant improvement in theoretical knowledge was observed at T1 relative to T0 (adjusted odds ratio [AOR] = 6.062; 95% CI: 2.946, 12.476; *p* < 0.001). However, by T2, retention had declined significantly: participants were less likely to pass the knowledge assessment compared with T0 (AOR = 0.486; *p* = 0.005). The main effect of group assignment was non-significant (*p* = 0.362), confirming baseline equivalence. Critically, the Group × Time interaction was non-significant at both T1 (AOR = 0.805; *p* = 0.657) and T2 (AOR = 1.553; *p* = 0.344), indicating that Repeated-Deliberate Practice did not yield a statistically significant benefit over a single session for long-term CPR knowledge retention.

**TABLE 3:** Effectiveness of the Repeated-DP group and Single-DP group on CPR knowledge level across time points (pre-training, immediately after training, 6 month follow up) adjusted with covariates.

Variables	Adjusted coefficient	Standard error	Adjusted odds ratio	95% CL for odds ratio		*P*-value
				Lower bound	Upper bound	
Intercept	1.317	2.225	–	–	–	–
Group						
Single-DP	Ref	–	–	–	–	–
Repeated-DP	−0.321	0.352	0.725	0.364	1.447	0.362
Time point						
T0	Ref	–	–	–	–	–
T1	1.802	0.368	6.062	2.946	12.476	<0.001*
T2	−0.722	0.260	0.486	0.292	0.807	0.005
Interaction (group × time)						
Single-DP × T0	Ref	–	–	–	–	–
Repeated-DP group × T1	−0.217	0.489	0.805	0.309	2.097	0.657
Repeated-DP group × T2	0.440	0.466	1.553	0.623	3.869	0.344

*Variable with *p* < 0.05. CI, confidence interval; Ref, reference category; QIC, 549.551; QICC, 543.100; T0, pre-training; T1, immediately after training; T2, retention (6-month follow up); Knowledge model adjusted with covariates: gender, age, BMI, education level, department, prior CPR experience.

### 3.4 Effectiveness of the Repeated-Deliberate Practice group versus the Single-DP group in sustaining CPR psychomotor skill retention across assessment time points (pre-training, immediately after training, and 6-month follow-up) adjusted with covariates

Table 4 presents the adjusted effect of Repeated-Deliberate Practice versus control on CPR skill retention at three time points: T0, T1, and T2, controlling for age, gender, BMI, educational background, department, and prior CPR experience. Prior CPR experience during actual cardiac arrest was a strong predictor of passing the skills test (AOR = 4.470; 95% CI: 1.602, 12.471; *p* = 0.004). Age, gender, BMI, educational background, and department were not statistically significant predictors (all *p* > 0.05).

**TABLE 4:** Effectiveness of the Repeated-DP group and control group on CPR skills retention across time points (pre-training, immediately after training, 6 month) adjusted with covariates.

Variables	Adjusted coefficient	Standard error	Adjusted odds ratio	95% CL for odds ratio		*P*-value
				Lower bound	Upper bound	
Intercept	2.110	0.374	–	–	–	–
Group						
Single-DP group	Ref	–	–	–	–	–
Repeated-DP group	−0.570	0.494	0.566	0.215	1.491	0.249
Time point						
T0	Ref	–	–	–	–	–
T1	−3.239	0.432	0.039	0.017	0.091	<0.001*
T2	−0.707	0.437	0.493	0.210	1.160	0.105
Interaction (group × time)						
Single-DP group × T0	Ref	–	–	–	–	–
Repeated-DP group × T1	1.667	0.560	5.294	1.765	15.880	0.003
Repeated-DP group × T2	−0.504	0.599	0.604	0.187	1.955	0.400

*Variable with *p* < 0.05. SE, standard error; CI, confidence interval; Ref, reference category; QIC, 477.259; QICC, 473.968; T1, immediately after training; T2, retention (6-month follow up). Knowledge model adjusted with covariates: gender, age, BMI, years of experience, education level, department, prior CPR experience.

Skills declined over time: failure rates at T1 were markedly lower than at T0 (AOR = 0.039; 95% CI, 0.015, 0.103; *p* < 0.001), indicating robust initial skill acquisition. By T2, however, performance in both groups had regressed to baseline levels (*p* = 0.105). The Group × Time interaction at T2 was non-significant (B = −0.504; *p* = 0.400), suggesting that a single 3-month booster session conferred no additional benefit for long-term psychomotor skill retention beyond initial training.

## 4 Discussion

This quasi-experimental study evaluated the effect of DP with varying retraining intervals on long-term retention of BLS competencies among novice nurses. As predicted by Deliberate Practice Theory, DP-based training yielded significant improvements in immediate skill acquisition and knowledge consolidation relative to baseline (*p* < 0.001). However, we found that, a 3-month DP booster session conferred no statistically significant improvement in long-term retention of BLS competencies.

Demographic analysis revealed no significant between-group differences in age, gender, BMI, or educational background, supporting baseline comparability. Critically, baseline CPR proficiency was uniformly low among newly graduated nurses: median chest compression depth and rate both fell significantly below the AHA guidelines for adult cardiopulmonary resuscitation—specifically, a depth of 50–60 mm and a rate of 100–120 compressions per minute. Only a small proportion of participants (7.43%) had performed CPR during actual cardiac arrest events. This finding corroborates prior evidence documenting a substantial “practice gap” between theoretical instruction and clinical readiness among novice nurses (30, 31). Novice nurses who fail to meet BLS clinical competence standards may compromise the resuscitation outcomes and patient safety of individuals experiencing in-hospital cardiac arrest. Therefore, targeted and evidence-informed CPR training programs should be prioritized for novice nurses.

In terms of theoretical knowledge, GEE analysis revealed a significant main effect of time (*p* < 0.001), with scores peaking immediately post-training and declining significantly by the 6-month retention assessment. The group × time interaction was non-significant (*p* = 0.32), indicating that the 3-month DP booster session did not meaningfully alter the trajectory of knowledge decay relative to the single-session condition. This pattern is consistent with Cognitive Load Theory, which posits that deliberate practice primarily enhances procedural skill acquisition and psychomotor automation, whereas declarative knowledge retention follows distinct temporal dynamics and may be more effectively sustained through spaced, multimodal reinforcement—such as brief digital learning modules or just-in-time cognitive aids—rather than isolated physical retraining sessions alone (32).

Similar to knowledge level, skill competency also declined markedly over time. Although both the Single-DP group and Repeated-DP group demonstrated high proficiency immediately after training, competency levels decreased substantially by the 6-month follow-up. Furthermore the outcomes of this study revealed no significant interaction effect at the 6-month follow-up (*p* = 0.400), indicating that an additional DP session at 3 months was also insufficient to prevent long-term skill decay. Our findings diverge from previous studies, such as those by Sutton et al. (33), Anderson et al. (21) which advocated that quarterly (3-month) training intervals were optimal for skill retention. While those studies often involved mixed cohorts of experienced and novice staff, our study focused exclusively on newly graduated nurses. Prior research demonstrated that nursing students exhibit significant declines in BLS knowledge as early as 3 months post-training, providing empirical support for the hypothesis of rapid attrition of both procedural skills and declarative knowledge (34). Therefore, a 3-month interval constitutes the critical threshold for psychomotor skill decay, and baseline competence directly informs the optimal reinforcement frequency for novice nurses.

For novices without prior CPR experience, a single 3-month booster session is insufficient to mitigate rapid psychomotor skill decay. The sparse reinforcement schedule impedes the consolidation of procedural memory—particularly for high-fidelity CPR competencies requiring precise chest compression depth, rate, and full recoil. Moreover, suboptimal training frequency undermines the long-term consolidation of procedural memory. Therefore, to promote the internalization of psychomotor skills and support their long-term consolidation into robust procedural memory, nurse training programs should adopt targeted learning strategies–particularly brief, high-frequency practice sessions—to strengthen retention and ensure guideline-concordant performance of CPR skills (17).

Notably, after adjusting for covariates, absence of prior clinical CPR experience emerged as a significant independent predictor of skill failure (*p* = 0.004). This finding underscores the protective role of prior hands-on CPR experience on the long-term maintenance of CPR skills. Crucially, we must address whether the consistency and stability of skills observed in simulation translate into clinically meaningful improvements during actual IHCA events. Standard simulation—even when repeated—often fails to fully replicate the cognitive and psychomotor reinforcement conferred by actual resuscitation events. This reflects a critical “authenticity gap,” wherein manikin-based practice lacks the physiological stressors, time-sensitive decisional complexity, and interprofessional coordination demands intrinsic to real-world resuscitation. While simulation proficiency is a recognized proxy for clinical readiness, our findings suggest that the “instability” observed in the simulation environment (i.e., rapid decay) likely predicts suboptimal performance at the bedside, potentially compromising chest compression fraction and hemodynamic quality during real arrests. Conversely, the stability demonstrated by nurses with prior clinical experience suggests that real-world stress inoculation facilitates a level of skill consolidation that creates a “transfer effect,” directly contributing to better patient outcomes. Therefore, bridging this gap requires more than just repetition; future curricula should incorporate high-fidelity simulations explicitly designed to emulate these authentic contextual features. By improving the stability of skills through high-fidelity or high-frequency training, we can increase the likelihood that these competencies will resiliently translate to clinically meaningful survival benefits for patients.

This study has several limitations. First, the quasi-experimental design—characterized by site-based allocation rather than individual-level randomization—introduces a risk of allocation bias. Although we adjusted for baseline demographic and professional characteristics in our analyses, residual confounding from unmeasured site-specific factors remains possible. Such factors may include variations in clinical workload, ward-level learning climates, or differences in leadership engagement across participating units—all of which could independently affect skill retention irrespective of the intervention. As a result, causal attribution to the intervention alone is necessarily constrained. To strengthen causal inference in future work, we recommend either (i) individual-level randomization, or (ii) multi-center cluster-randomized trials with hierarchical modeling (e.g., mixed-effects or GEE approaches) explicitly accounting for site-level clustering and contextual heterogeneity.

## 5 Conclusion

This study demonstrates that DP training significantly enhances BLS skills among novice nurses, a population shown to exhibit consistently suboptimal chest compression performance during CPR. These findings underscore the need for systematic reform in clinical training programs. Specifically, clinical educators should replace traditional single-session CPR training with high-frequency, high-fidelity simulation, short-interval DP-based practice to promote durable skill retention and progressive improvement in clinical performance. Such an approach is essential for bridging the gap between theoretical knowledge and real-world application, ultimately ensuring higher standards of care and improved patient safety in the management of critically ill patients.

## Data Availability

The original contributions presented in this study are included in this article/Supplementary material, further inquiries can be directed to the corresponding authors.
