# Supplementary material for: Effects of deliberate practice at different training frequencies on long-term retention of high-quality chest compression proficiency among novice nurses: a quasi-experimental study
Source: Front Med (Lausanne). 2026 Apr 10;13:1751992. doi: 10.3389/fmed.2026.1751992 (PMC13106074; doi:10.3389/fmed.2026.1751992)
Supplement: Supplementary file 1 [file Supplementary_file_1.docx]

**Supplementary Material 1**

**Theoretical Assessment Questions**

1. What is the most common cause of cardiac arrest in adults?

A. Cardiac problems

B. Congenital or acquired cardiac defects

C. Respiratory failure or shock

D. Infections and sepsis

2. When you witness a patient collapse and become unresponsive, and you are the first to arrive at the scene, what should be your initial action?

A. Activate the emergency response system

B. Begin high-quality cardiopulmonary resuscitation (CPR), starting with chest compressions

C. Initiate rescue breaths

D. Ensure the scene is safe for both yourself and the patient

3. When should an automated external defibrillator (AED) be used upon arrival at the scene?

A. After approximately 2 minutes of 5 cycles of CPR

B. During the pause between every 30 chest compressions and 2 rescue breaths

C. Immediately upon availability

D. Only after professional medical personnel arrive, and then handed over to them

4. What are the recommended rate and depth for chest compressions in adult patients during CPR?

A. More than 100 compressions per minute, approximately 5 cm

B. More than 100 compressions per minute, 5–6 cm

C. 100–120 compressions per minute, 5–6 cm

D. 100–120 compressions per minute, approximately 5 cm

5. Which of the following is NOT one of the five key components of high-quality CPR?

A. Rapid and forceful chest compressions

B. Complete chest recoil

C. Avoidance of excessive ventilation

D. Early recognition

6. Which of the following statements regarding the compression-to-ventilation ratio is incorrect?

A. Adult, single rescuer: 15:2

B. Adult, two rescuers: 30:2

C. Child, single rescuer: 30:2

D. Child, two rescuers: 15:2

7. Which of the following is NOT a recommended sign for laypersons to identify cardiac arrest?

A. Unresponsiveness

B. Absence of breathing

C. Agonal gasps

D. Absence of carotid pulse

8. What is the correct sequence for CPR (A: Airway, B: Breathing, C: Circulation)?

A. A-B-C

B. C-A-B

C. C-B-A

D. B-A-C

9. The correct location for chest compressions during CPR is:

A. Upper half of the sternum

B. Lower half of the sternum

C. Middle portion of the sternum

D. Just above the xiphoid process

10. For patients without cervical spine injury, what is the preferred technique for manual airway opening?

A. Head-tilt/chin-lift maneuver

B. Jaw-thrust maneuver

C. Head-tilt/neck-lift maneuver

D. Tongue-jaw lift maneuver

11. For patients suspected of having cervical spine injury, what is the preferred technique for manual airway opening?

A. Head-tilt/chin-lift maneuver

B. Jaw-thrust maneuver

C. Head-tilt/neck-lift maneuver

D. Tongue-jaw lift maneuver

12. What is the correct sequence for operating an automated external defibrillator (AED)?

A. Power on → Place electrodes → Analyze rhythm → Charge → Deliver shock

B. Place electrodes → Power on → Analyze rhythm → Deliver shock

C. Place electrodes → Power on → Analyze rhythm → Charge → Deliver shock

D. Power on → Place electrodes → Analyze rhythm → Deliver shock

13. What is the most rapid and effective intervention for cardiac arrest?

A. Mouth-to-mouth artificial ventilation

B. External chest compression

C. Internal cardiac massage

D. External chest compression combined with artificial ventilation

14. Which of the following is an indication for asynchronous direct current defibrillation?

A. Sinus arrest

B. Atrial fibrillation

C. Supraventricular tachycardia

D. Ventricular fibrillation

15. What is the most common rhythm observed in witnessed, non-traumatic out-of-hospital cardiac arrest?

A. Asystole

B. Pulseless electrical activity

C. Ventricular fibrillation

D. Electrical-mechanical dissociation

16. What is the simplest and most reliable method to assess the effectiveness of external chest compressions during cardiopulmonary resuscitation?

A. Palpation of the carotid pulse

B. Palpation of the radial pulse

C. Observation of pupillary response

D. Assessment of consciousness

17. After how long without adequate cerebral perfusion does the brain typically sustain irreversible damage during cardiac arrest?

A. 2–3 minutes

B. 4–6 minutes

C. 7–8 minutes

D. More than 10 minutes

18. What is the recommended ventilation rate per minute for a patient with an advanced airway in place during ongoing CPR?

A. 6–8 breaths per minute

B. 12–16 breaths per minute

C. 16–20 breaths per minute

D. 10 breaths per minute

19. When initiating cardiopulmonary resuscitation in a patient confirmed to be in cardiac arrest, what should be performed first?

A. Airway management

B. Rescue breathing

C. Chest compressions

D. Precordial thump

20. Which of the following is NOT considered an appropriate technique for performing high-quality chest compressions?

A. The shoulder, elbow, and wrist joints should be aligned in a straight line

B. The hips should serve as the fulcrum for compression force

C. The heel of the hand must remain in firm contact with the sternum without lifting or shifting between compressions

D. The primary force should be generated solely by the arms

**Supplementary Material 2**

**The Adult Basic Life Support (BLS) Structured Skill Checklist**

| Name： |  | Sex： | Date： | |  | |
| --- | --- | --- | --- | --- | --- | --- |
| content | Operational requirements | | | | Score | Score |
| Assess the environment  (3 points) | The rescuer observes the surrounding environment to it is safe. | | | | 1 |  |
|  | Take good personal protective measure | | | | 1 |  |
|  | Check the watch and record the time of resuscitation | | | | 1 |  |
| Assess consciousness  (3 points) | Pat both shoulders and call both ears  (0.5 points will deducted for calling to only one ear) | | | | 1 |  |
|  | The assessment time: 3-5 seconds | | | | 1 |  |
|  | The rescuer and patient are in the correct positions | | | | 1 |  |
| Assess breathing and pulse ( 5 points） | Open the patient's clothes | | | | 1 |  |
|  | Assess breathing | | | | 1 |  |
|  | Correctly locate the pulse position | | | | 1 |  |
|  | Judgment time: 5 to 10 seconds | | | | 1 |  |
|  | Place on a hard floor | | | | 1 |  |
| Call and start EMS（3 points） | Call for assistant | | | | 1 |  |
|  | The assistant prepares the bag-valve mask. | | | | 1 |  |
|  | Prepare the defibrillator | | | | 1 |  |
| External cardiaccompression (30 points) | Complete 30 compression in 15 to 18 seconds | Location (1 point will be deducted for each mistake) | | | 2 |  |
|  |  | Pressing position (0.4 points deducted for each mistake) | | | 2 |  |
|  |  | Objective score: Pressing qualification rate * total score | | Rate (100-120 /min) | 20 |  |
|  |  |  |  | Depth (50~60mm) |  |  |
|  |  |  |  | Chest recoil |  |  |
|  |  | Objective scoring: Compression score (<60%: 0 points, >60%: 2 points) | | | 2 |  |
|  |  | Pay attention to observing the patient's complexion (0.4 points for each cycle) | | | 2 |  |
|  |  | Compressions to maintaining a 30:2 compression to ventilation ratio (minus 0.4 scores per mistake) | | | 2 |  |
| Inspection and evaluation  (4 points) | The heartbeat and breathing resumed, and the cardiopulmonary resuscitation was successful. | | | | 1 |  |
|  | Place the patient in the recovery position. | | | | 1 |  |
|  | Sort out the clothes and prepare for transfer. | | | | 1 |  |
|  | Take off the gloves, wash hands and record the time when the rescue ends. | | | | 1 |  |
| Open airway, and perform artificial ventilation（20 points） | Check the mouth for any foreign objects. | | | | 1 |  |
|  | The method of pressing the forehead and lifting the chin is correct. | | | | 1 |  |
|  | E-C technique (1 point will be deducted for each mistake)Maintain an open airway continuously. | | | | 5 |  |
|  | Maintain an open airway continuously. (1 point will be deducted for each mistake. | | | | 3 |  |
|  | Objective score: Ventilation qualification rate *Total score | The chest rises and falls. | | | 10 |  |
|  |  | Ventilation time ≥1 second | | |  |  |
| Overall evaluation  （6 points） | Standardized movements | | | | 2 |  |
|  | Proficient in the process | | | | 2 |  |
|  | Have a perfect understanding of each other's intentions | | | | 2 |  |
| The total operation time should be less than 4 minutes (1 point will be deducted for every 5 seconds exceeded). | | | | |  |  |
| **Overall score** | | | | |  | |
| **Instructor name** | | | | |  | |
